# Analysis of the Transcriptional Control of *Bcl11b* in Chicken: IRF1 and GATA1 as Negative Regulators

**DOI:** 10.3390/ani15050665

**Published:** 2025-02-25

**Authors:** Lingling Qiu, Haojie Wang, Wenhao Li, Ting Yang, Hao Bai, Guobin Chang

**Affiliations:** 1 College of Animal Science and Technology, Yangzhou University, Yangzhou 225009, China; 007621@yzu.edu.cn (L.Q.); hjwang2024@163.com (H.W.); l2823667541@163.com (W.L.); yt1144736744@163.com (T.Y.); 2 Joint International Research Laboratory of Agriculture and Agri-Product Safety the Ministry of Education of China, Institutes of Agricultural Science and Technology Development, Yangzhou University, Yangzhou 225009, China; bhowen1027@yzu.edu.cn

**Keywords:** *Bcl11b*, promoter, transcriptional regulation, ALV-J, chicken

## Abstract

B-cell lymphoma/leukemia 11B (Bcl11b) plays roles in cell proliferation and apoptosis and holds a pivotal position within the immune system. Our previous studies have demonstrated that Bcl11b can promote cell apoptosis to curb ALV-J infection. To gain insights into the molecular mechanisms underlying *Bcl11b* expression regulation in chickens, we constructed various truncated dual luciferase reporter vectors and analyzed the promoter region of *Bcl11b*. We employed promoter-binding TF profiling assay and the dual luciferase assay of site-directed mutagenesis and the expression level of interfering or overexpressing transcription factors were used to study their transcriptional regulation mechanism of chicken *Bcl11b* and functions in ALV-J infection. Our findings revealed core regulatory regions of the chicken *Bcl11b* promoter. By examining the −606~−363 bp region, we identified several transcription factors and their binding sites. Mutational and functional analysis further revealed interferon regulatory factor-1 (IRF1) and GATA-binding protein 1 (GATA1) as critical factors for the repression of chicken *Bcl11b*, thereby affecting cell apoptosis and ALV-J replication. Furthermore, DNA methylation analysis indicated that methylation may also contribute to changes in *Bcl11b* promoter activity. These findings offer valuable insights into the regulatory mechanisms of chicken *Bcl11b* and provide promising targets for molecular breeding and genetic improvement of disease resistance in chickens.

## Simple Summary

The *Bcl11b* gene is important for cell growth and death in chickens. Understanding how Bcl11b helps combat a virus known as avian leukosis virus subgroup J (ALV-J) is essential. Researching the part of the gene that controls its activity revealed that two proteins, IRF1 and GATA1, play a key role in controlling *Bcl11b*. These proteins affect how cells die and how much virus is made. The study also showed that DNA methylation can influence its activity. This research provides valuable information for improving chicken resistance to diseases, which can be beneficial for the poultry industry and food safety.

## 1. Introduction

Avian leukosis virus subgroup J (ALV-J) can cause multiple tumors, stunted growth, and reduced egg production in chickens [1,2]. It spreads quickly through horizontal (feces and fluids) and vertical transmission [3,4,5]. Despite its severe economic impact, no effective medications or vaccines exist to control ALV-J infection. Consequently, the discovery of potent disease-resistance factors or regulatory targets holds potential as a promising approach to bolstering resistance breeding efforts and enhancing animal disease resistance.

In the interaction between viruses and their hosts, viral replication is often influenced by host factors. Previous reports have identified PMAIP1 [6], SOCS3 [7], TCP1 [8], SHP-2 [9], DOT1L [10], etc., as promoters of viral replication, while IRF7 [11], TRIM62 [12], CH25H [13], etc., inhibit viral replication. Additionally, specific receptors such as chNHE1, serving as the receptor for ALV-J, have emerged as important targets for biological disease-resistant breeding [14,15]. However, a critical gap remains: most studies on host–virus interactions have focused on individual genes or receptors (e.g., chNHE1), but these approaches risk being circumvented by viral evolution [16]. To achieve durable and broad-spectrum resistance, it is essential to target upstream regulatory loci and mechanisms rather than individual genes or receptors.

Our previous investigations have shown that chicken Bcl11b promotes cellular apoptosis while simultaneously suppressing the replication of ALV-J [17,18]. *Bcl11b* encodes a Krüppel-like C2H2 zinc finger transcription factor that binds to DNA, either repressing or activating transcription, thereby playing a vital role in gene expression [19,20,21]. Prior research has uncovered the role of Bcl11b in regulating development, proliferation, and differentiation, highlighting its significance in the immune system, blood-related disorders, neurodevelopmental conditions, and neuropsychiatric diseases [22,23,24]. Bcl11b is indispensable at various stages of T-cell precursor differentiation and plays a pivotal role in the proliferative response of peripheral CD8^+^ T-cells against viruses, intracellular bacteria, and tumors [21,25]. Studies on HIV have also shown that Bcl11b can inhibit HIV transcription [26]. Notably, the zinc finger domain located at the N-terminus of Bcl11b exhibits typical CCHC zinc finger structural characteristics, and this domain has been implicated in antiviral activities [27]. Researchers have uncovered that Bcl11b modulates Marek’s disease virus replication [28]. microRNAs (miRNAs) have been reported to regulate *Bcl11b* translation [29,30,31,32,33]. Additionally, transcription factors can bind to promoter sequences to either activate or inhibit gene transcription [34,35]. While Bcl11b’s antiviral role is established, its transcriptional regulation mechanism—specifically, how its expression is activated or repressed—remains unknown. Consequently, it is crucial to investigate the regulatory mechanisms controlling *Bcl11b*.

In this study, by mapping the core promoter region of chicken *Bcl11b* and identifying its key transcription suppressors, we clarify how Bcl11b expression is controlled and validate these regulators as potential targets for ALV-J resistance breeding.

## 2. Materials and Methods

### 2.1. Ethics Statement

All experiments were conducted at Yangzhou University and animal experiments were approved by the Institutional Animal Care and Use Committee of Yangzhou University (approval number: 202103358).

### 2.2. Isolation and Culture of Cells

Chicken embryo fibroblast (CEF) cells were isolated from 10-day-old White Leghorns specific-pathogen-free (SPF) chicken embryos (Poultry Institute, Shandong Academy of Agricultural Science, Jinan, Shandong, China). These cells were cultivated in Dulbecco’s modified Eagle medium (DMEM) (Hyclone, Logan, UT, USA) supplemented with 10% fetal bovine serum (FBS) (Gibco, Grand Island, NY, USA) and 1% glutamine (Gibco).

The chicken fibroblast cell line (DF-1) was acquired from the American Type Culture Collection (ATCC) (Manassas, VA, USA) (Catalog Number: CRL-3586). The DF-1 cells were maintained in DMEM medium, supplemented with 10% FBS and 1% L-glutamine. All cells were incubated at a temperature of 37 °C under an atmosphere of 5% CO_2_ and maintained at a relative humidity range from 60–70%.

### 2.3. ALV-J Infection and Validation

The ALV-J strain (GY03) was kindly provided by Professor Qian (the College of Veterinary Medicine, Yangzhou University, Yangzhou, Jiangsu, China). Cells were infected with ALV-J in serum-free medium at a concentration of 10^4^ TCID_50_ per 0.1 mL and then incubated for 2 h at 37 °C with 5% CO_2_ with gentle shaking every 30 min. Cells were also mock-infected using serum-free medium under the same incubation conditions. Following this, the infected cells were maintained in serum-starved environment for the indicated time.

To confirm virus infection, we conducted immunofluorescence assays (IFA) using a specific protocol. First, cells were fixed in 4% paraformaldehyde for 8 min. Following this, the cells were rinsed three times with phosphate-buffered saline (PBS) (Hyclone) and blocked using a blocking buffer for 30 min. The cells were then incubated overnight at 4 °C with a mouse monoclonal antibody, JE9, specifically targeting the ALV-J gp85 protein (diluted to 1:2000), which was kindly provided by Professor Qin. After three PBS washes, the cells were incubated for 1 h at room temperature with fluorescein isothiocyanate (FITC)-labeled goat anti-mouse IgG antibody (Santa Cruz, Dallas, TX, USA). Subsequently, the cells were stained with 2-(4-amidinophenyl)-6-indolecarbamidine dihydrochloride (DAPI; Beyotime, Shanghai, China), mounted using antifade mounting medium (Beyotime), and finally observed under an Olympus SP70 fluorescence microscope (Olympus Corporation, Tokyo, Japan).

### 2.4. Bioinformatics Analysis of Promoter

We obtained the upstream sequences of *Bcl11b*, spanning 2199 bp and identified by Gene ID NC_052536, from the NCBI (National Center for Biotechnology Information) website: https://www.ncbi.nlm.nih.gov/ (accessed on 4 June 2023). To analyze this region, we employed several online prediction tools: BDGP (Neural Network Promoter Prediction): https://fruitfly.org/seq_tools/promoter.html (accessed on 4 June 2023), the Promoter 2.0 Prediction Server found at http://www.cbs.dtu.dk/servic-es/Promoter/ (accessed on 4 June 2023), FPROM [36] available at http://www.softberry.com/berry.phtml?topic=fprom&group=programs&subgroup=promoter (accessed on 4 June 2023), and TSSW located at http://www.softberry.com/berry.phtml?topic=tssw&group=programs&subgroup=promoter (accessed on 4 June 2023). Additionally, we acquired 2999 bp sequences upstream of *Bcl11b* for 27 species, chicken included, from NCBI. Using Molecular Evolutionary Genetics Analysis (MEGA) software version 7.0, a versatile tool for sequence alignment, phylogenetic tree inference, database mining, molecular evolution rate estimation, and evolutionary hypothesis testing, we analyzed the homology of these sequences and constructed phylogenetic trees.

For the prediction and analysis of potential transcription factor binding sites within the *Bcl11b* promoter region, we utilized both the JASPAR CORE database [37] accessible at https://jaspar.elixir.no/ (accessed on 24 July 2024) and AliBaba 2.1 found at http://gene-regulation.com/pub/programs/alibaba2/index.html (accessed on 24 July 2024).

### 2.5. Vectors Construction

Chicken blood was used to extract genomic DNA using the TIANamp Genomic DNA Kit (TIANGEN, Beijing, China) following the manufacturer’s guidelines. Polymerase chain reaction (PCR) was then performed to obtain truncated promoter fragments, using PrimeSTAR^®^ GXL DNA Polymerase (Takara, Tokyo, Japan) according to the manufacturer’s instructions. The following PCR cycling profile was used: one single step at 98 °C for 10 s, followed by 35 cycles of 98 °C for 10 s, 60 °C for 15 s, 68 °C 10 s/kb, and ending with 68 °C for 5 min. The resulting PCR product was purified with the MiniBEST Agarose Gel DNA Extraction Kit (Takara) and subsequently subcloned into the Sac I and Hind III (NEB, Ipswich, MA, USA) digested pGL3-Basic vector (Promega, Beijing, China). The plasmid was then extracted using the EndoFree Midi Plasmid Kit (TIANGEN). Sanger sequencing was conducted to verify all cloned plasmids. The primers employed for vector construction are listed in Table S1. Additionally, fragments of the *Bcl11b* promoter region containing mutations in the putative binding sites were cloned and inserted into the multiple cloning site (MCS) of the pGL3-Basic Luciferase Report Vector, the wild and mutant type plasmids sequence are shown in Table S2. For preparation of the unmethylated pGL3-708+19 vector, the plasmid pGL3-708+19 was amplified in the dam−/dcm− *E. coli* HST04 strain (Takara).

RNA was extracted from spleen tissues or cells using TRIzol reagent (ThermoFisher Scientific, Waltham, MA, USA). High-quality chicken spleen cDNA was synthesized using the PrimeScript™ 1st Strand cDNA Synthesis Kit (Takara) for the construction of an overexpression vector. The coding sequences (CDS) were amplified by PCR (PCR reaction conditions were consistent with those mentioned above) and subcloned into the Kpn I and Xho I digested pcDNA 3.1 vector (resulting in pcDNA3.1-IRF1 and pcDNA3.1-GATA1). The primers used for the overexpression vector construction are provided in Table S3.

### 2.6. siRNA Design and In Vitro Transcription

The target sites of *GATA1* and *IRF1* CDS sequences were designed using an online tool: http://biodev.extra.cea.fr/DSIR/DSIR.html (accessed on 25 December 2024), DNA templates were synthesized by Tsingke Biotech (Beijing, China) and the target sequences and negative control (NC) are listed in Table S4. Transcription and purification were performed in vitro according to instructions of the T7 RNAi Transcription Kit (Vazyme, Nanjing, China).

### 2.7. Transfection and Dual Luciferase Assay

The activities of truncated promoter fragment plasmids, both wild-type and mutant plasmids, were evaluated using a dual-luciferase reporter assay. Briefly, DF-1 cells were plated at a density of approximately 7 × 10^4^ cells per well in 24-well plates and allowed to grow overnight prior to transfection. Following the protocol outlined in the Lipofectamine™ 3000 Transfection Reagent manual (ThermoFisher Scientific, MA, USA), the cells were co-transfected with the plasmid pRL-TK (Promega). After 24 h, the luciferase activities were assessed using the Dual-Luciferase^®^ Reporter Assay System (Promega), in which Renilla luciferase activity was used as an internal control to normalize the data and correct for variations in transfection efficiency and sample loading. The experiment was conducted with three biological replicates, and each sample was analyzed to ensure accuracy.

### 2.8. Promoter-Binding Transcription Factor Profiling Assay

Briefly, nuclear protein was extracted from CEF cells using the NE-PER™ Nuclear and Cytoplasmic Extraction Reagents Kit (ThermoFisher), and the concentration was measured with a BCA Protein Assay Kit (Beyotime), following the manufacturer’s guidelines. Promoter-binding transcription factor profiling assay was conducted with Promoter-Binding TF Profiling Plate Array I (Signosis, Santa Clara, CA, USA). The reaction mixture, comprising TF binding buffer, probes, nuclear protein, and either the *Bcl11b* promoter fragment or no DNA, was incubated at room temperature for 30 min. Subsequently, free probes were separated, and bound probes were eluted, hybridized to the plate, and incubated overnight at 42 °C. Detection of bound probes involved using an HRP–streptavidin conjugate with a chemiluminescent substrate, and the resulting luminescence was quantified using EnSpire Multimode Plate Reader (PerkinElmer, Boston, MA, USA).

### 2.9. Western Blot

Cell lysates were prepared using RIPA buffer supplemented with 1 mM PMSF (Beyotime). Subsequently, protein concentrations were measured using the Enhanced BCA Protein Kit (Beyotime). SDS-PAGE was employed to separate the total proteins, which were then transferred onto polyvinylidene difluoride (PVDF) membranes (Bio-Rad, Shanghai, China) that had been activated with methanol. Following membrane blocking, the PVDF membranes were incubated overnight at 4 °C with primary antibodies diluted to a recommended ratio. These membranes were then further incubated with their respective secondary antibodies. Imaging of the membranes was carried out using the FluorChem FC3 Universal imaging system (ProteinSimple, San Jose, CA, USA), and the protein expression levels were quantitatively analyzed with the assistance of Image-J 20 software (NIH, Bethesda, MD, USA).

### 2.10. ALV-J Intervention Study

DF-1 cells were plated in 12-well plates at a density of approximately 2 × 10^5^ cells per well and allowed to grow overnight prior to ALV-J infection. Subsequently, the cells were infected with ALV-J in serum-free medium at a dose of 10^4^ TCID_50_/0.1 mL. The infection process involved incubating the cells for 2 h at 37 °C with 5% CO_2_, while gently shaking them at 30 min intervals. Following infection, the cells were maintained in serum starvation culture for an additional 24 h. At 24 hpi, the cells were transfected with overexpression vectors or siRNAs. Cell samples were then collected 48 h after transfection for assessment of virus replication.

### 2.11. qRT-PCR Analysis

After synthesizing cDNA using HiScript III RT SuperMix for qPCR (+gDNA wiper) (Vazyme), we conducted quantitative real-time polymerase chain reaction (qRT-PCR) analysis using ChamQ Blue Universal SYBR qPCR Master Mix (Vazyme) on QuantStudio 3 real-time PCR system (ThermoFisher Scientific). The primers were designed through Primer-BLAST: http://www.ncbi.nlm.nih.gov/tools/primer-blast/ (accessed on 14 July 2024) or selected from previously validated primers used in our earlier experiments, and synthesized by Tsingke Biotech (Beijing, China). Primer efficiencies were determined using a 10-fold serial dilution of plasmid DNA or cDNA. The amplification efficiencies were close to 100%, which is considered acceptable for reliable qPCR analysis. Gene expression levels were calculated using the 2^−ΔΔCt^ method. The following PCR cycling profile was used: one single step at 95 °C for 30 s, followed by 40 cycles of 95 °C for 10 s and 60 °C for 30 s, ending with a melting curve analysis of 95 °C for 15 s, 60 °C for 1 min, 95 °C for 15 s and 60 °C for 15 s. To ensure accuracy, two reference genes, *GAPDH* and *SDHA*, served as internal controls. Each assay was performed in triplicate, and the specific primer sequences are listed in Table S3.

At the specified time points, we assessed the relative abundance of virus. The cells were collected, and total RNA was extracted from them using TRIzol reagent (Life Technologies, Carlsbad, CA, USA) following the standard protocol. Specifically, we designed primers of ALV-J (*Env*) for qRT-PCR to assess viral loads in ALV-J-infected cells (Table S3).

### 2.12. Cell Apoptosis

Cells were collected and subsequently assessed for apoptosis using the Annexin V-FITC Apoptosis detection kit (Vazyme, Nanjing, China). Briefly, the harvested cells underwent two washes with cold PBS, followed by a 10 min incubation in the dark at room temperature with both annexin V-FITC and propidium iodide (PI). Apoptosis in these cells was then evaluated through a FACS Fortessa flow cytometer (BD Biosciences, San Jose, CA, USA). The resulting flow cytometric data was analyzed utilizing FlowJo software (V10, FlowJo, Palo Alto, CA, USA).

### 2.13. Cell Proliferation Assay

Cell proliferation was assayed using the Cell Counting Kit-8 (CCK-8, Vazyme), adhering to the manufacturer’s guidelines. Approximately 48 h post-transfection, the CCK-8 reagent was introduced to the cells and allowed to incubate for a period ranging from 0.5 to 2 h. Subsequently, the optical density at 450 nm of each well was measured utilizing the Infinite M2000 Pro instrument (Tecan, Männedorf, Switzerland).

### 2.14. BSP Methylation Sequencing

Genomic DNA was extracted from cells both with and without ALV-J infection. Subsequently, this DNA underwent bisulfite treatment, which specifically converts unmethylated cytosine (C) residues to uracil (U), leaving methylated cytosines unchanged. Following purification of the bisulfite-converted DNA, PCR amplification was performed. The resultant PCR products were then cloned into sequencing vectors. Subsequently, the sequencing results were compared and analyzed to assess the methylation status of CpG sites.

### 2.15. Statistical Analysis

Statistical analyses of data were conducted using GraphPad Prism 8 software (GraphPad Software Inc., San Diego, CA, USA). The results were presented as the mean ± SEM. *p* < 0.05 was deemed to indicate a statistically significant difference.

## 3. Results

### 3.1. Promoter Region Analysis of Chicken Bcl11b Gene

**Figure 1:**
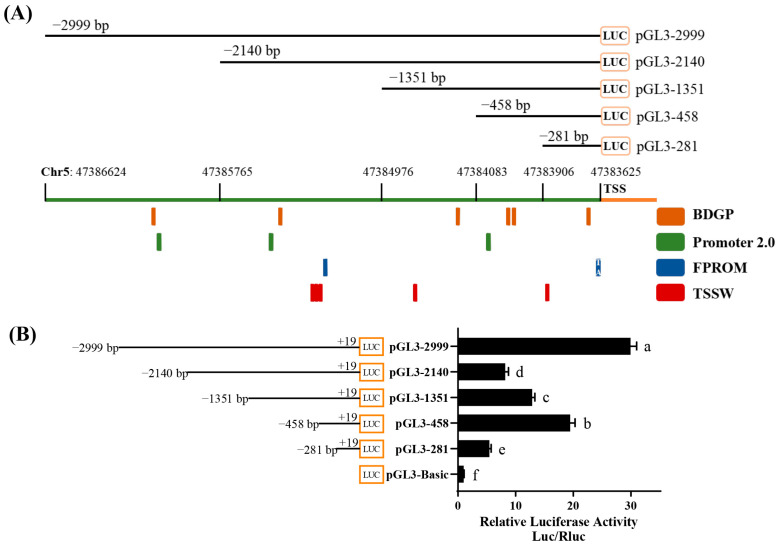
Promoter analysis of Chicken *Bcl11b* gene. (**A**) Online prediction of *Bcl11b* promoter region. (**B**) Dual luciferase activity assay with various truncated fragments from the upstream region of TSS of the chicken *Bcl11b* gene. Mean ± SEM, different letters (a–f) represent significant differences between groups at a significance level of *p* < 0.05.

To gain further insights into the regulatory mechanisms controlling *Bcl11b* expression, we employed various promoter prediction tools to analyze the −2999 bp sequence upstream of the transcription start site (TSS) of chicken *Bcl11b* (Figure 1A). Based on this, we cloned various truncated fragments from the upstream region of chicken *Bcl11b* TSS into a dual luciferase reporter vector (pGL3-Basic). These constructs were co-transfected into DF-1 cells with pRL-TK. To assess transcriptional activity, we measured changes in luciferase activity 48 h after transfection. The pGL3-Basic vector was used as the control plasmid and pRL-TK as an internal reference plasmid. As depicted in Figure 1B, the pGL3-2999 exhibited the highest relative luciferase activity. Notably, deletions of the −2999~−2140 bp and −458~−281 bp regions led to significantly reduced transcriptional activity. Conversely, removing the −2140 to −458 bp region gradually recovered transcriptional activity. These preliminary findings suggest that the *Bcl11b* promoter can be divided into three functional regulatory regions. Specifically, the −2140 to −458 bp region contains significant negative regulatory elements, whereas the −2999 to −2140 bp and −458 to −281 bp regions harbor positive regulatory elements. Our results offer valuable insights into the transcriptional regulatory mechanisms of *Bcl11b* in chickens.

### 3.2. Bioinformatics Analysis of the Promoter Region of Bcl11b

**Figure 2:**
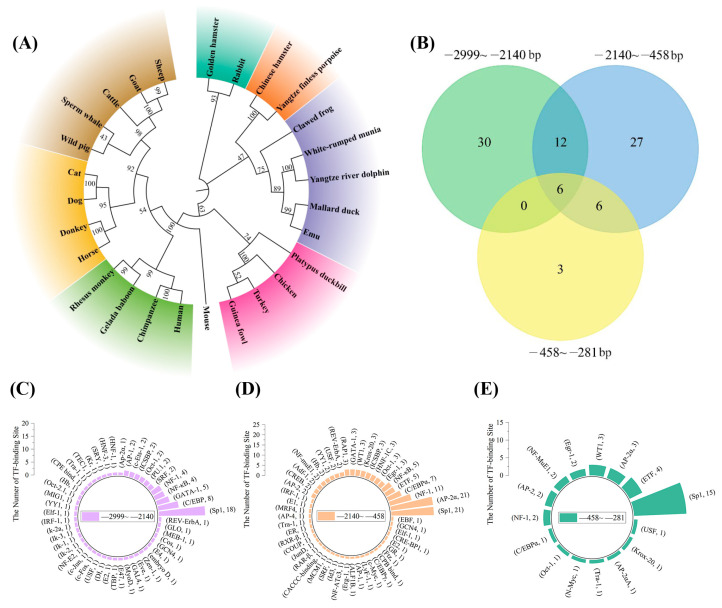
Bioinformatics analysis and visualization of the *Bcl11b* promoter region. (**A**) Phylogenetic tree of *Bcl11b* promoter sequence (−2999~+19 bp) of 27 species. The same color represents groups that are clustered together. (**B**) Venn diagram analysis to visualize the set relationships of predicted transcription factors across different regulatory regions of chicken *Bcl11b* promoter. (**C**–**E**) Binding predictions for transcription factors across three regulatory regions of chicken *Bcl11b* promoter. Each individual bar corresponds to one transcription factor, with the bar length indicative of the number of binding sites.

We conducted a phylogenetic and evolutionary analysis of the *Bcl11b* promoter region across 27 species. Utilizing the maximum likelihood estimation (ML) method available in MEGA 7 and Chiplot online website (https://www.chiplot.online/ (accessed on 15 June 2024)) [38], we constructed a phylogenetic tree that revealed a close relationship between the chicken *Bcl11b* promoter region and those of turkeys and guinea fowl, with a notable lack of homology to the promoter regions of other species (Figure 2A). Given that transcription factors play a crucial role in regulating gene expression by binding to promoter sequences, we further analyzed the *Bcl11b* promoter regions using AliBaba 2.1: http://gene-regulation.com/pub/programs/alibaba2/ (accessed on 24 July 2024) to predict the transcription factors for three distinct regions: −2999 ~ −2140 bp, −2140~−458 bp and −458~−281 bp (Figure 2B). To identify potential regulatory elements that enhance or inhibit gene transcription, we employed Venn analysis to visualize the relationships among transcription factors in these different regulatory regions (summarized in Table S5). Additionally, we counted the number of transcription factor binding sites within each regulatory region, enabling us to calculate the frequency of motifs and present these findings in Figure 2C–E. Notably, our analysis revealed that Sp1, a ubiquitously expressed transcription factor, exhibits the highest frequency of binding to the chicken *Bcl11b* promoter, followed by Ap-2α, NF-1, C/EBPα, and other transcription factors.

### 3.3. Identification of Core Regions and Key Transcription Factors of Chicken Bcl11b Promoter

**Figure 3:**
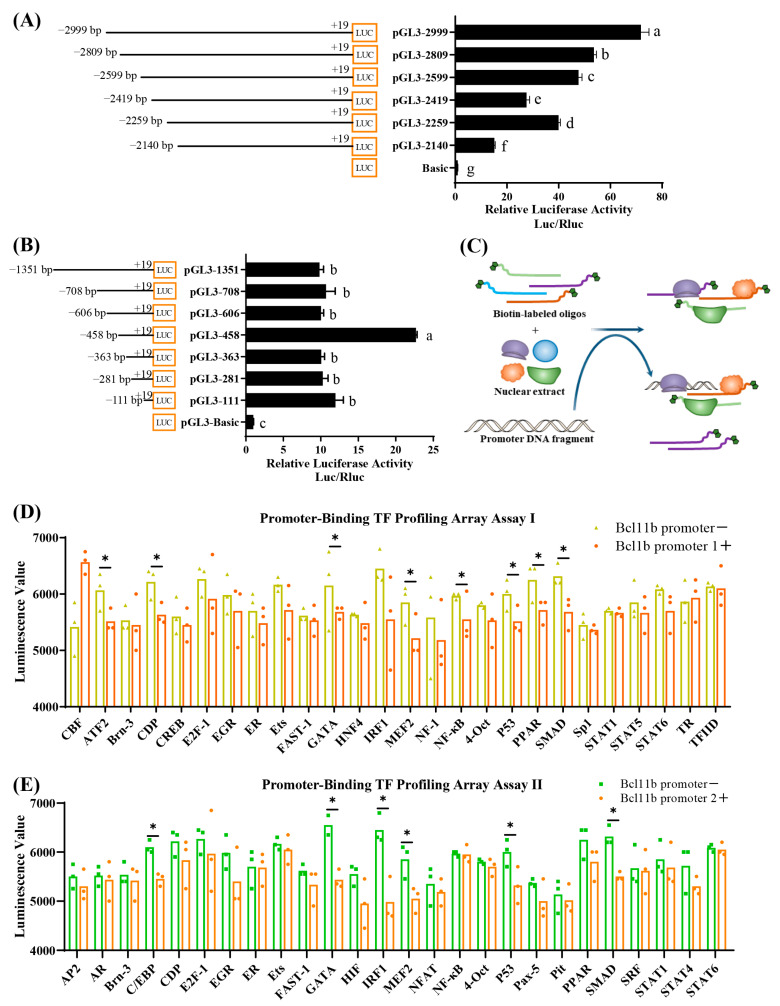
Identification of core regulatory regions and crucial transcription factors of the chicken *Bcl11b* promoter. (**A**,**B**) A dual luciferase reporter assay was conducted using different truncated fragments spanning three regulatory regions of the chicken *Bcl11b* as described earlier. (**C**) A schematic representation of the profiling assay for TFs binding to the promoter. (**D**) Identification of TFs that bind to the region spanning from −2259 to −2140 bp of the chicken *Bcl11b* promoter. (**E**) Identification of TFs that bind to the region spanning from −606 to −363 bp of the chicken *Bcl11b* promoter. Data are present as mean ± SEM, with different letters (a–g) indicating statistically significant differences between groups (*p* < 0.05). * for *p* < 0.05.

To delve deeper into the core regions involved in transcriptional activity and to pinpoint potential transcription factors, we truncated the three aforementioned regulatory regions to different lengths and observed how these alterations affected transcriptional activity, as indicated by changes in luciferase activity. As depicted in Figure 3A,B, deletion of −2259~−2140 bp and −458~−363 bp region notably decreased promoter activity, suggesting the presence of significant positive regulatory elements within these regions. Conversely, removing the region of −606~−458 bp significantly increased transcriptional activity, indicating the existence of negative regulatory elements within this region of *Bcl11b*. In summary, the core promoter regions for the chicken *Bcl11b* encompass the regions of −2259~−2140 bp and −606~−363 bp.

To identify the primary transcriptional regulatory elements that interact with two core promoter regions of chicken *Bcl11b*, we conducted a promoter-binding TF profiling assay. The underlying concept of this assay is that the core promoter regions competitively bind with transcription factors, leading to a decrease in the number of biotin-labeled probes bound to these factors, which is reflected by lower luminescence readings (Figure 3C). By comparing the luminescence values in the presence and absence of the *Bcl11b* core promoter regions, we can pinpoint the crucial regulatory elements (Figure 3D,E). Specifically, Figure 3D reveals that when the −2259 to −2140 bp region of the *Bcl11b* promoter is present, the luminescence values for ATF2, CDP, MEF2, NF-κB, P53, PPAR, and SMAD are notably lower than when this promoter fragment is absent. The preliminary data suggests that the first core region (−2259~−2140 bp) of chicken *Bcl11b* promoter harbors regulatory elements for ATF2, CDP, MEF2, NF-κB, P53, PPAR, and SMAD. Analogously, Figure 3E shows that the luminescence values for C/EBP, GATA, IRF1, MEF2, P53, and SMAD are significantly higher in the absence of the −606~−363 bp region of the *Bcl11b* promoter compared to when this region is included. These preliminary findings indicate that the second core region (−606~−363 bp) of the chicken *Bcl11b* promoter contains essential regulatory elements for C/EBP, GATA, IRF1, MEF2, P53, and SMAD.

### 3.4. Detection and Confirmation of Key Transcription Factors Binding Sites Within the Second Core Region of the Chicken Bcl11b Promoter

Previous research has revealed that chicken Bcl11b suppresses ALV-J replication. We delved deeper into the binding sites and functional role of transcription factors within the second core region of the *Bcl11b* promoter and expected to identify transcription factors and their specific binding sites.

**Figure 4:**
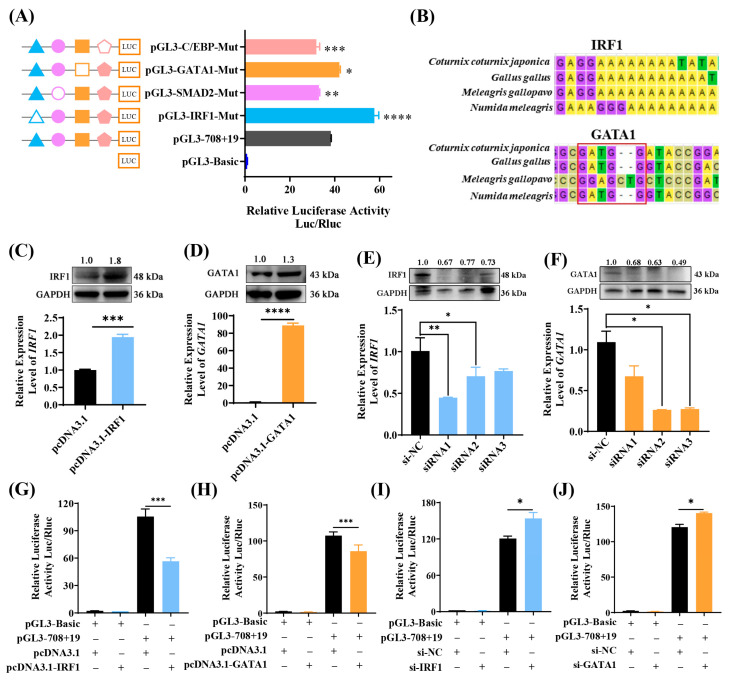
Detection and confirmation of key transcription factors binding sites within the second core region of the chicken *Bcl11b* promoter. (**A**) Dual luciferase activity test with site-directed mutations at the C/EBPβ, GATA1, SMAD2, and IRF1 sites in the pGL3-708+19 vector, using pGL3-708+19 as a comparison. (**B**) Alignment of multiple sequences focusing on the IRF1 and GATA1 transcription factor binding sites in the *Bcl11b* promoter across species. The red box indicates GATA1 transcription factor binding site. (**C**,**D**) Overexpression of chicken IRF1 and GATA1, with the pcDNA3.1(+) empty vector serving as negative control. (**E**,**F**) Interference of chicken IRF1 and GATA1 expression, with the siRNA NC serving as negative control. (**G**,**H**) Luciferase reporter assessments following IRF1 or GATA1 overexpression, achieved through co-transfection with either the pGL3-708+19 vector in DF-1 cells. (**I**,**J**) Luciferase reporter assessments following IRF1 or GATA1 knockdown, achieved through co-transfection with either the pGL3-708+19 vector in DF-1 cells. The results are presented as Mean ± SEM. Compared to the control group, significance levels are indicated by asterisks: * for *p* < 0.05, ** for *p* < 0.01, *** for *p* < 0.001, and **** for *p* < 0.0001.

The JASPAR CORE database was employed to predict transcription factors and their respective binding sites within the −606~−363 bp region of the *Bcl11b* promoter. By amalgamating these predictions with the results from the promoter-binding TF profiling assay, it was revealed that the mentioned region contains binding sites for IRF1, SMAD2, C/EBPβ, and GATA1. The motifs of these transcription factors and their corresponding binding sites are illustrated in Figures S1 and S2. To confirm the existence and functional relevance of these transcription factor binding sites within the second core region of chicken *Bcl11b* promoter, plasmids containing site-directed mutations for these transcription factors were constructed within the pGL3−708+19 vector and transfected into chicken DF-1 cells (Figure 4A). The outcomes demonstrated that mutations targeting the SMAD2 and C/EBPβ binding sites in pGL3−708+19 vectors led to a significant decrease in transcriptional activity, whereas mutations at the IRF1 and GATA1 sites notably enhanced transcriptional activity. Consequently, it is hypothesized that SMAD2 and C/EBPβ function as positive regulators to promote chicken *Bcl11b* transcription, whereas IRF1 and GATA1 may serve as negative regulators, inhibiting the transcription of chicken *Bcl11b*. Additionally, we examined the conservation of IRF1 and GATA1 transcription factors across various species, such as quail (*Coturnix coturnix japonic*), chicken (*Gallus gallus*), turkey (*Meleagris gallopavo*), and guineafowl (*Numida meleagris*) (Figure 4B) and found that the binding sites for IRF1 and GATA1 are highly conserved across these species. These findings provide the rationale for further exploration of their pivotal roles in regulating the transcription of chicken *Bcl11b*.

To further ascertain the involvement of IRF1 and GATA1 in modulating chicken *Bcl11b* promoter activity, we constructed overexpression vectors for IRF1 and GATA1, validated them at both the mRNA and protein levels (Figure 4C,D), and then co-transfected them into chicken DF-1 cells along with pGL3−708+19 vector. Luciferase activity was assayed 48 h post-transfection. The outcomes demonstrated that overexpression of IRF1 and GATA1 markedly suppressed the activity of the *Bcl11b* core promoter (Figure 4G,H). Concurrently, we designed multiple siRNAs targeting the *IRF1* and *GATA1*, transcribed them in vitro, and validated their interference efficacy at both the mRNA and protein levels (Figure 4E,F). Subsequently, the siRNA exhibiting the most effective interference was selected and co-transfected into DF-1 cells with the pGL3-708+19 vector. As shown in Figure 4I,J, knockdown of IRF1 and GATA1 resulted in a notable increase in the activity of the *Bcl11b* core promoter.

These findings imply that transcription factors IRF1 and GATA1 inhibit the transcriptional activity of chicken *Bcl11b* by binding to its promoter core region, thereby playing pivotal roles in regulating chicken *Bcl11b* expression.

### 3.5. IRF1 and GATA1 Suppress Bcl11b Transcription and Promote ALV-J Replication

**Figure 5:**
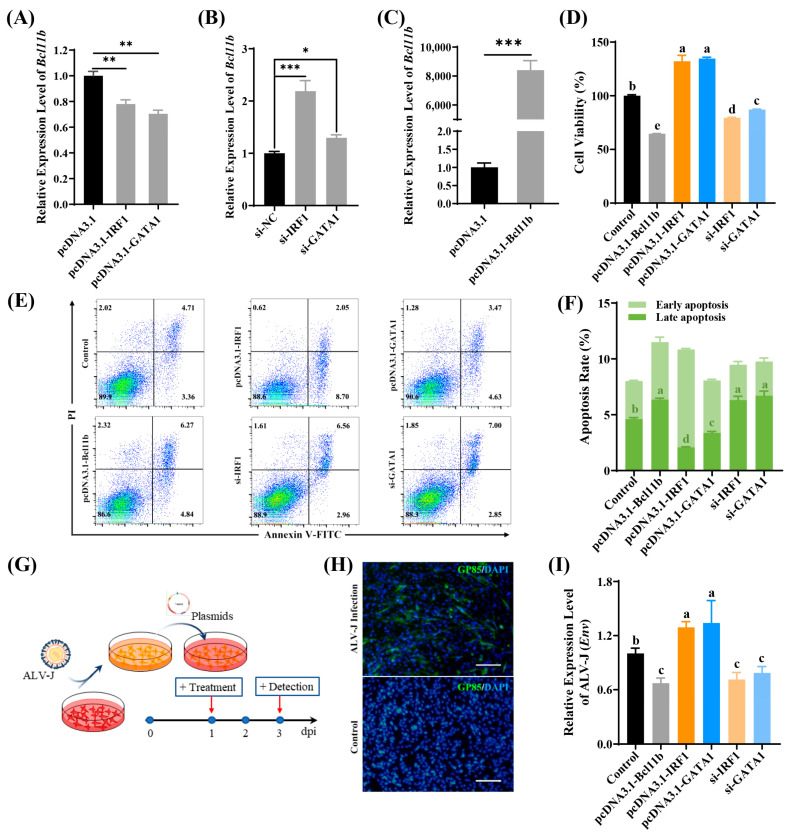
IRF1 and GATA1 suppress *Bcl11b* transcription and promote ALV-J replication. (**A**,**B**) The expression levels of *Bcl11b* after we altered GATA1 and IRF1 expression, respectively. (**C**) The expression levels of *Bcl11b*, the pcDNA3.1(+) empty vector was used as negative control. (**D**) When Bcl11b, GATA1, and IRF1 expressions were modified, changes in cell viability were noted. (**E**,**F**) Changes in cell apoptosis when *Bcl11b*, *GATA1*, and *IRF1* expressions were altered. (**G**) A detailed procedure for intervening in ALV-J-infected cells. (**H**) Immunofluorescence staining was conducted using an antibody specific to the viral protein gp85 to detect ALV-J virus infection (green). Nuclear staining was achieved using DAPI (Blue). The scale bar indicates 50 μm. (**I**) The impact on ALV-J replication was assessed when Bcl11b, GATA1, and IRF1 expressions were altered. Data are presented as mean ± SEM. Compared to the control group, * indicates significance at *p* < 0.05, ** indicates significance at *p* < 0.01, and *** indicates significance at *p* < 0.001. Different letters (a–e) denote significant differences between groups at *p* < 0.05.

To gain a deeper understanding of the role of IRF1 and GATA1 in regulating chicken *Bcl11b* expression and their impact on ALV-J replication, we analyzed their expression patterns in DF-1 cells infected with ALV-J. As depicted in Figure S3, upon infection with ALV-J, there was a notable upregulation in the expressions of *GATA1* and *IRF1* within 24 h, followed by a subsequent decrease at 36 h post-infection (hpi). This suggests that *GATA1* and *IRF1* are involved in ALV-J infection. Subsequently, we individually overexpressed GATA1 and IRF1 and measured the *Bcl11b* expression level in DF-1 cells (Figure 5A). At the same time, we also sequentially knocked down the expression of GATA1 and IRF1 to detect their effects on the expression of *Bcl11b* in DF-1 cells (Figure 5B). The findings revealed that overexpressing both GATA1 and IRF1 suppressed the expression of chicken *Bcl11b*, whereas knockdown of both GATA1 and IRF1 significantly promoted chicken *Bcl11b* expression. This further confirms their negative regulatory roles in controlling *Bcl11b* expression.

Our previous research revealed that chicken Bcl11b is pivotal in modulating apoptosis and proliferation and exhibiting antiviral function. Subsequently, we delved into the roles of GATA1 and IRF1 in cell apoptosis, proliferation, and ALV-J replication. Results presented in Figure 5C,D demonstrate that overexpression of *Bcl11b* significantly inhibits cell viability, whereas overexpression of GATA1 or IRF1 alone significantly enhances cell viability. When *IRF1* or *GATA1* expression is knocked down, cell viability is suppressed. As shown in Figure 5E,F, overexpression of *Bcl11b* significantly increases the level of cell apoptosis, whereas overexpression of GATA1 or IRF1 alone inhibits late apoptosis. When GATA1 or IRF1 expression is knocked down, the level of late apoptosis increases significantly. Based on these previous results, it is speculated that GATA1 and IRF1 affect cell viability and apoptosis by regulating the expression of *Bcl11b*.

We further investigated the roles of IRF1 and GATA1 on ALV-J replication. We individually altered the expression levels of IRF1, GATA1, and Bcl11b 24 h post-infection with ALV-J. Subsequently, we evaluated the viral replication levels after an additional 48 h (Figure 5G). As shown in Figure 5H, cells infected with ALV-J were identified with monoclonal antibodies specifically targeting the ALV-J virus-specific protein gp85, with no fluorescence observed in the negative control group. Figure 5I illustrates that overexpression of *Bcl11b* notably suppressed ALV-J replication, whereas overexpression of IRF1 and *GATA1* significantly increased ALV-J replication. Conversely, when IRF1 or GATA1 expression was knocked down, the level of ALV-J replication was markedly inhibited.

These findings suggest that the transcription factors IRF1 and GATA1 suppress the expression of chicken *Bcl11b* by binding to its promoter region, counteract apoptosis, and prolong cell survival, ultimately facilitating cell apoptosis and the replication of ALV-J.

### 3.6. Methylation Analysis of the Second Core Region of the Chicken Bcl11b Promoter

**Figure 6:**
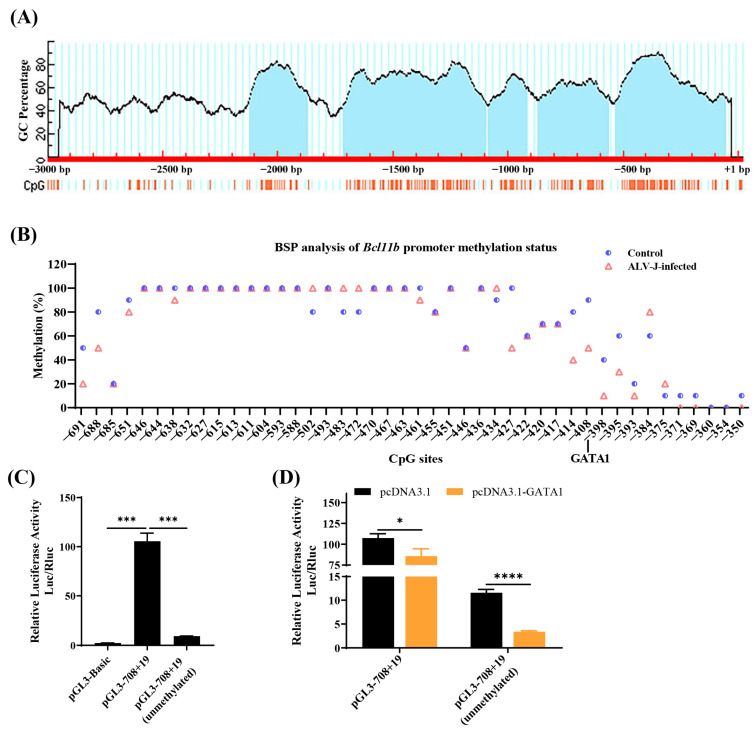
Methylation analysis of the second core region of the chicken *Bcl11b* promoter. (**A**) CpG island in the entire *Bcl11b* promoter predicted by METHPRIMER. (**B**) Methylation of the second core promoter region of chicken *Bcl11b*. (**C**) DNA demethylation represses the *Bcl11b* promoter activity. (**D**) DNA demethylation in the second core promoter region of *Bcl11b* enhances the inhibition of promoter activity mediated by negative regulatory elements such as GATA1. Data are presented as mean ± SEM. Compared to the control group, * indicates significance at *p* < 0.05, *** indicates significance at *p* < 0.001, and **** indicates significance at *p* < 0.0001.

According to predictions by Methprimer, multiple CpG islands exist within the *Bcl11b* promoter (Figure 6A). The second core promoter region contains a typical CpG island with 71 CpG sites. To investigate the methylation status of this second core promoter region, bisulfite sequencing (BS) was conducted, revealing that most of the CpG islands in the second core promoter region are highly methylated, with no significant changes observed after ALV-J infection. However, the methylation status of CpG sites at and near the GATA1 element binding site significantly decreased following ALV-J infection (Figure 6B). It is speculated that methylation at some CpG sites and GATA1 regulatory element binding sites may also contribute to changes in *Bcl11b* promoter activity.

To test this hypothesis, we first conducted a dual luciferase reporter to determine whether DNA methylation affects *Bcl11b* promoter activity. The reporter gene analysis in Figure 6C indicates that the activity of the unmethylated pGL3-708+19 is extremely significantly lower than that of the normally methylated pGL3-708+19. The reporter gene analysis in Figure 6D shows that GATA1 extremely significantly inhibits the activity of the unmethylated pGL3-708+19, reducing its activity by 50% compared to the normally methylated pGL3-708+19. These results suggest that the removal of methylation strengthens the inhibitory effect of negative regulatory elements (e.g., GATA1) on promoter activity.

## 4. Discussion

**Figure 7:**
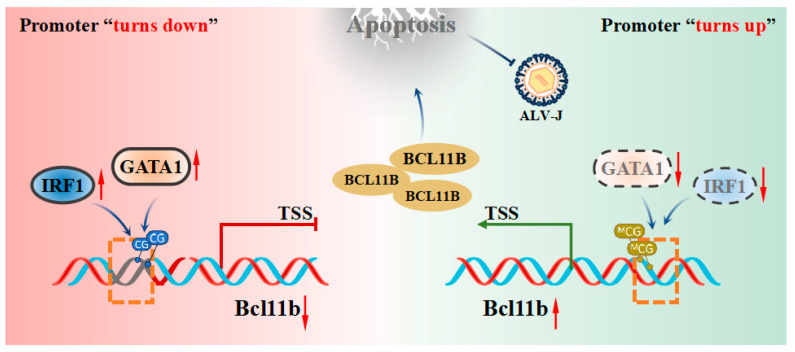
Schematic diagram of how transcription factor IRF1 and GATA1 regulate the transcription of chicken *Bcl11b* and thereby affect cell apoptosis and ALV-J replication. The orange boxes represent transcription factor binding sites.

Previous studies have demonstrated that chicken *Bcl11b*, functioning as an interferon-stimulated gene, encodes the C2H2-type zinc finger protein Bcl11b, which promotes cell apoptosis and inhibits ALV-J replication [17,18,21,39]. Interestingly, *Bcl11b* expression is suppressed in the majority of ALV-J-infected individuals. However, the regulatory elements governing its transcription—particularly the transcription factors responsible for its activation or repression—remain poorly characterized [34,35]. Our study identifies IRF1 and GATA1 as key transcriptional suppressors of *Bcl11b* and facilitating ALV-J replication (Figure 7).

In this study, we obtained the promoter sequence of the chicken *Bcl11b* and identified its core promoter regions. Consistent with numerous studies, we predicted that Sp1, a widely expressed transcription factor, would bind frequently to chicken *Bcl11b* promoters [40]. We also discovered numerous other potential transcription factors, including C/EBP, GATA, IRF1, MEF2, P53, and SMAD. However, the transcription factors identified in this study are different from those identified in mice, such as Notch signaling, GATA-3, and TCF1) [41,42,43], which were not predicted in chicken *Bcl11b*. This discrepancy may be attributed to the limited homology between mouse and chicken promoters.

Our findings revealed that IRF1 and GATA1 regulatory elements markedly suppressed *Bcl11b* transcription in chickens by binding to the *Bcl11b* promoter. This suppression subsequently affected cell viability, apoptosis, and ALV-J replication. Xie et al. also found that IRF1 represses target gene expression in humans by interfering with the activation of other transcription factors, which is consistent with the inhibitory role of IRF1 observed in our study [44]. In addition, IRF1 is known for its instability, with its expression temporarily increasing in response to external stimuli before undergoing rapid degradation, which is consistent with what we observed in ALV-J-infected DF-1 cells [45,46]. However, numerous studies have also reported that IRF1 can activate gene transcription by binding to the promoter regions of the target genes [47,48,49], which is inconsistent with our findings. It may hinge on factors such as post-translational modifications, promoter context, or the intracellular environment [50]. Additionally, GATA1 has been reported in the regulation of natural immune signaling pathways in mice [51]. It has been reported that it promotes the replication of the B19 virus in HEK293 cells, which is consistent with our results [52]. This conservation suggests that GATA1 may be a universal target for viral exploitation. Research in human breast cancer cells also identified GATA1 as a negative regulatory factor on the target gene [53], which is consistent with what we observed in our study. The negative regulatory effect of IRF1 and GATA1 likely occurs through competitive binding to the *Bcl11b* promoter, displacing activators or recruiting co-repressors.

Furthermore, we also found that demethylation in the *Bcl11b* promoter represses promoter activity which may be mediated by negative regulatory elements such as GATA1. Commonly, DNA methylation is viewed as inhibiting the expression of neighboring genes. Yet, there have also been reports indicating that DNA methylation can, in some cases, stimulate gene expression [54,55,56]. A study has revealed that DNA methylation partially enhances *PPARγ* expression by blocking NRF1 from binding to the gene promoter, aligning with our observations [57]. Similarly, the Polycomb protein binds the *FoxA2* promoter, suppressing its expression. Promoter methylation blocks this binding, upregulating *FoxA2* expression [58]. The role of methylation in *Bcl11b* regulation likely depends on the interplay between specific transcription factors and chromatin modifiers, emphasizing the need for further mechanistic studies.

The identification of IRF1 and GATA1 as negative regulators of chicken *Bcl11b* provides potential targets for genetic editing (e.g., CRISPR/Cas9) to enhance ALV-J resistance in chickens. It also provides strategies to enhance *Bcl11b* expression which could serve as a complementary approach to combat ALV-J infection. While our study provides novel insights into the regulation of *Bcl11b* and its role in ALV-J infection, several limitations should be acknowledged: our study focuses on transcriptional and epigenetic regulation, *Bcl11b* activity is likely modulated by additional layers, including histone modifications, m6A RNA methylation, and post-translational modifications. Future studies are needed to explore the roles and regulatory mechanisms mediated by histone acetylation/m6A modifications of chicken Bcl11b during ALV-J infection. Furthermore, conducting further research to assess the feasibility of the binding sites involved in this study as targets for disease-resistant breeding is necessary.

## 5. Conclusions

In conclusion, we have shown that the transcription factors IRF1 and GATA1 negatively regulate chicken *Bcl11b* transcription, which reduces Bcl11b-mediated cell apoptosis and enhances ALV-J replication. Additionally, DNA methylation blocks inhibitory elements (GATA1) to indirectly enhance *Bcl11b* transcription. Our findings deepen our understanding of the transcriptional regulatory mechanisms controlling chicken *Bcl11b* and offer potential targets for precision breeding strategies to enhance ALV-J resistance in poultry.

## Data Availability

All datasets generated or analyzed during this study are available from the corresponding author upon reasonable request.

## Supplementary Materials

The following supporting information can be downloaded at: https://www.mdpi.com/article/10.3390/ani15050665/s1, Figure S1: Transcription factor binding motifs; Figure S2: The predicted transcription factor binding sites in chicken Bcl11b promoter; Figure S3: The expression pattern of GATA1 and IRF1 in DF-1 cells infected with ALV-J. Table S1: The primers information for promoter cloning; Table S2: Wild and mutant type plasmids sequences; Table S3: The primers information for overexpression, qRT-PCR; Table S4: Target sequence of siRNAs; Table S5: Venn analysis of predicted transcription factors in different regulatory regions.

## Abbreviations

The following abbreviations are used in this manuscript:

Bcl11b	B-cell lymphoma/leukemia 11B
ALV-J	Avian leukosis virus subgroup J
IRF1	Interferon regulatory factor-1
GATA1	GATA-binding protein 1
TSS	Transcription start site
siRNA	Small Interfering RNA
hpi	Hours post-infection
