# Supplementary material for: Analysis of the Transcriptional Control of Bcl11b in Chicken: IRF1 and GATA1 as Negative Regulators
Source: Animals (Basel). 2025 Feb 25;15(5):665. doi: 10.3390/ani15050665 (PMC11898421; doi:10.3390/ani15050665)
Supplement: Supplementary file 1 [file animals-15-00665-s001.zip › animals-3470409-supplementary.pdf]

Supplementary Material

Analysis of the Transcriptional Control of *Bcl11b* in Chicken: IRF1 and GATA1 as Negative Regulators

Lingling Qiu [1], Haojie Wang [1], Wenhao Li [1], Ting Yang [1], Hao Bai [2] and Guobin Chang [1,*]

Supplementary Figure S1-S3

Supplementary Table S1-S5

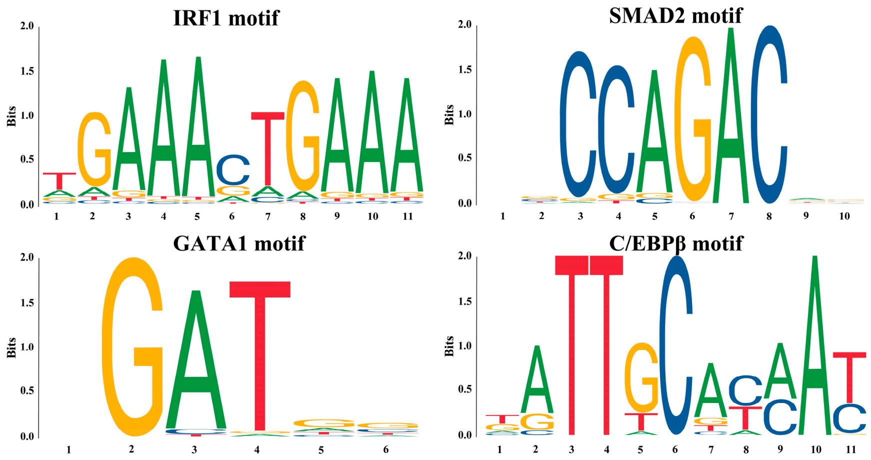

Figure S1 Transcription factor binding motifs.

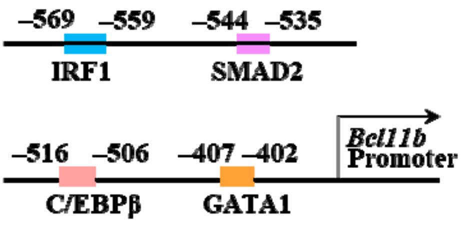

Figure S2 The predicted transcription factor binding sites in chicken Bcl11b promoter.

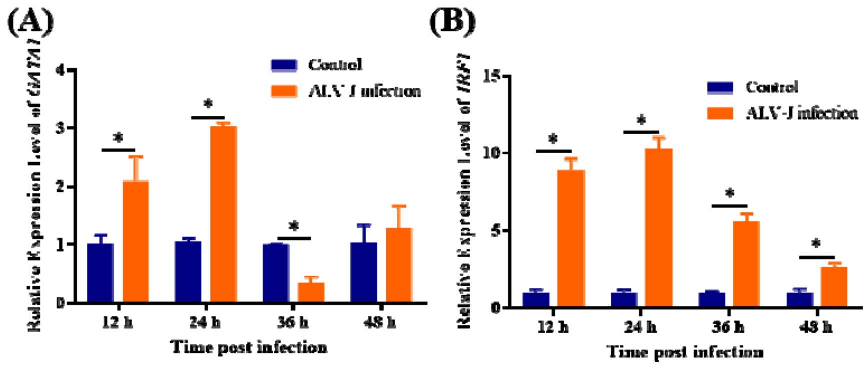

Figure S3 The expression pattern of GATA1 and IRF1 in DF-1 cells infected with ALV-J.

Table S1. The primers information for promoter cloning

Name of Plamids	Primer Sequence (5′-3′)	Length/bp
pGL3-2999	F: tatcgataggtaccgagctcAAGGTGGAGCGGCTAACG	3018
pGL3-2140	F: tatcgataggtaccgagctcATAAGGAGCAGCGACTCCCGG	2159
pGL3-1351	F: tatcgataggtaccgagctcGGCTTCGTGAGAGCCAAAGC	1370
pGL3-708	F: tatcgataggtaccgagctcTCCTCCCAGAGAAACCCTCGC	727
pGL3-458	F: tatcgataggtaccgagctcGGGACGGGCGGCC	477
pGL3-363	F: tatcgataggtaccgagctcCCCCGGCCCCGAG	382
pGL3-281	F: tatcgataggtaccgagctcGGATGCGGCTTTGTGTGTCC	300
pGL3-111	F: tatcgataggtaccgagctcGTGCGGTGTGTGCGAGT	130
Bcl11b-Hind III	R: ccggaatgccaagcttCGGACGTGACGTTTAATCTGCAC	-
pGL3-2809	F: tatcgataggtaccgagctcCTCTCTCCTCTCCCCCC	2828
pGL3-2599	F: tatcgataggtaccgagctcATCGGGACTTCCTCTTCACG	2618
pGL3-2419	F:tatcgataggtaccgagctcCATCAGCCTGTTTTCCCCTATGACA	2438
pGL3-2259	F:tatcgataggtaccgagctcCAAGGCACAAACAGCAAATAGCAC	2278
pGL3-606	F: tatcgataggtaccgagctcGTGTCCCGCTGCCATCA	625
Bcl11b-Nhe I	R: tcgagcccgggctagcCGGACGTGACGTTTAATCTGCAC	-

Note: The lower-case letters are overlap sequences, underlined letters are enzyme cutting sites.

Table S2. Wild and mutant type plasmids sequences

Plasmids	Promoter sequences
pGL3-708+19	GAGCTCTCCTCCCAGAGAAACCCTCGCCGCCGGGATGTAAGGAAAA<br>GGGGTGGGAAAGGAGGGTCGGAGCGCGTGTGCGTGTGCGGCTCGG<br>GAATGTGTGCGCGCGTGTCCCGCTGCCATCACGCTTCGCTTTAGGGA<br>AAGAGGAGGAAAAAAAAAAAATTCTCTCCCCAACCCACACAGATAG<br>TGGTGGTGGTGGTGGTGGTGTAAAACTGTCGGAAGAAACGCAGGGG<br>GACGAGGAGCTGCCGCGGCGTCCGCGGGGACGGGCGGCCCGAGCC<br>CCTCCGCGGGGAGCGCACCGCGGCGCCGGGGGCGATGGGTACCGAC<br>GCGGCCCCACCGCAGCCCCCGCCCGCGCCTCCCCCGGCCCCGAGCG<br>GCCCCCGGCAGCCGCCCCCGGCACAGCCGCCCCCGCCGCGGCGCGG<br>GGAAGTTTGGCCCCGCGTGAGGATGCGGCTTTGTGTGTCCCCCCCTC<br>AAATGCTCTTTGCTTCGCTCCCAGCGCTCCGCGTACCCGAGAAAGCG<br>GCAGCAGCGCACAAAGCCCGGAGCTGCTCCCGATCCGTGCATGTGCT<br>TTAGTTTGTGTCTGTGTGTGTGTGTGTATGTGTGTGTGTGTGTGAGTG<br>AGTGCGGTGTGTGCGAGTGGCGTTTCTTGTTCTCTTGCAGGGTACAA<br>TGTTAAAAAGCCACCGCTAGTCGCCCCCAGTGCTCCTACTCTCTGGG<br>TCTTTTTGTCTCTAGTGCAGATTAAACGTCACGTCCGACGCGT
pGL3-C/EBPβ-Mut	GAGCTCTCCTCCCAGAGAAACCCTCGCCGCCGGGATGTAAGGAAAA<br>GGGGTGGGAAAGGAGGGTCGGAGCGCGTGTGCGTGTGCGGCTCGG<br>GAATGTGTGCGCGCGTGTCCCGCTGCCATCACGCTTCGCTTTAGGGA<br>AAGAGGAGGAAAAAAAAAAAATTCTCTCCCCAACCCACACAGATAG<br>TGGTGGTGGTGGTGCTGTCGGAAGAAACGCAGGGGGACGAGGAGCT<br>GCCGCGGCGTCCGCGGGGACGGGCGGCCCGAGCCCCTCCGCGGGGA<br>GCGCACCGCGGCGCCGGGGGCGATGGGTACCGACGCGGCCCCACCG<br>CAGCCCCCGCCCGCGCCTCCCCCGGCCCCGAGCGGCCCCCGGCAGC<br>CGCCCCCGGCACAGCCGCCCCCGCCGCGGCGCGGGGAAGTTTGGCC<br>CCGCGTGAGGATGCGGCTTTGTGTGTCCCCCCCTCAAATGCTCTTTGC<br>TTCGCTCCCAGCGCTCCGCGTACCCGAGAAAGCGGCAGCAGCGCAC<br>AAAGCCCGGAGCTGCTCCCGATCCGTGCATGTGCTTTAGTTTGTGTCT<br>GTGTGTGTGTGTGTATGTGTGTGTGTGTGTGAGTGAGTGCGGTGTGT<br>GCGAGTGGCGTTTCTTGTTCTCTTGCAGGGTACAATGTTAAAAAGCC<br>ACCGCTAGTCGCCCCCAGTGCTCCTACTCTCTGGGTCTTTTTGTCTCT<br>AGTGCAGATTAAACGTCACGTCCGACGCGT
pGL3-GATA1-Mut	GAGCTCTCCTCCCAGAGAAACCCTCGCCGCCGGGATGTAAGGAAAA<br>GGGGTGGGAAAGGAGGGTCGGAGCGCGTGTGCGTGTGCGGCTCGG<br>GAATGTGTGCGCGCGTGTCCCGCTGCCATCACGCTTCGCTTTAGGGA<br>AAGAGGAGGAAAAAAAAAAAATTCTCTCCCCAACCCACACAGATAG<br>TGGTGGTGGTGGTGGTGGTGTAAAACTGTCGGAAGAAACGCAGGGG<br>GACGAGGAGCTGCCGCGGCGTCCGCGGGGACGGGCGGCCCGAGCC<br>CCTCCGCGGGGAGCGCACCGCGGCGCCGGGGGGTACCGACGCGGCC<br>CCACCGCAGCCCCCGCCCGCGCCTCCCCCGGCCCCGAGCGGCCCCC<br>GGCAGCCGCCCCCGGCACAGCCGCCCCCGCCGCGGCGCGGGGAAGT

	TTGGCCCCGCGTGAGGATGCGGCTTTGTGTGTCCCCCCCTCAAATGC<br>TCTTTGCTTCGCTCCCAGCGCTCCGCGTACCCGAGAAAGCGGCAGCA<br>GCGCACAAAGCCCGGAGCTGCTCCCGATCCGTGCATGTGCTTTAGTT<br>TGTGTCTGTGTGTGTGTGTGTATGTGTGTGTGTGTGAGTGAGTGCG<br>GTGTGTGCGAGTGGCGTTTCTTGTTCTCTTGCAGGGTACAATGTTAAA<br>AAGCCACCGCTAGTCGCCCCCAGTGCTCCTACTCTCTGGGTCTTTTTG<br>TCTCTAGTGCAGATTAAACGTCACGTCCGACGCGT
pGL3-SMAD2-Mut	GAGCTCTCCTCCCAGAGAAACCCTCGCCGCCGGGATGTAAGGAAAA<br>GGGGTGGGAAAGGAGGGTCGGAGCGCGTGTGCGTGTGCGGCTCGG<br>GAATGTGTGCGCGCGTGTCCCGCTGCCATCACGCTTCGCTTTAGGGA<br>AAGAGGAGGAAAAAAAAAAAATTCTCTCCCCAATAGTGGTGGTGGT<br>GGTGGTGGTGTAAAACTGTCGGAAGAAACGCAGGGGGACGAGGAG<br>CTGCCGCGGCGTCCGCGGGGACGGGCGGCCCGAGCCCCTCCGCGGG<br>GAGCGCACCGCGGCGCCGGGGGCGATGGGTACCGACGCGGCCCCAC<br>CGCAGCCCCCGCCCGCGCCTCCCCCGGCCCCGAGCGGCCCCCGGCA<br>GCCGCCCCCGGCACAGCCGCCCCCGCCGCGGCGCGGGGAAGTTTGG<br>CCCCGCGTGAGGATGCGGCTTTGTGTGTCCCCCCCTCAAATGCTCTTT<br>GCTTCGCTCCCAGCGCTCCGCGTACCCGAGAAAGCGGCAGCAGCGC<br>ACAAAGCCCGGAGCTGCTCCCGATCCGTGCATGTGCTTTAGTTTGTG<br>TCTGTGTGTGTGTGTGTATGTGTGTGTGTGTGAGTGAGTGCGGTGT<br>GTGCGAGTGGCGTTTCTTGTTCTCTTGCAGGGTACAATGTTAAAAAG<br>CCACCGCTAGTCGCCCCCAGTGCTCCTACTCTCTGGGTCTTTTTGTCT<br>CTAGTGCAGATTAAACGTCACGTCCGACGCGT
pGL3-IRF1-Mut	GAGCTCTCCTCCCAGAGAAACCCTCGCCGCCGGGATGTAAGGAAAA<br>GGGGTGGGAAAGGAGGGTCGGAGCGCGTGTGCGTGTGCGGCTCGG<br>GAATGTGTGCGCGCGTGTCCCGCTGCCATCACGCTTCGCTTTAGGGA<br>AAGAGGAAAATTCTCTCCCCAACCCACACAGATAGTGGTGGTGGTGG<br>TGGTGGTGTAAAACTGTCGGAAGAAACGCAGGGGGACGAGGAGCTG<br>CCGCGGCGTCCGCGGGGACGGGCGGCCCGAGCCCCTCCGCGGGGAG<br>CGCACCGCGGCGCCGGGGGCGATGGGTACCGACGCGGCCCCACCGC<br>AGCCCCCGCCCGCGCCTCCCCCGGCCCCGAGCGGCCCCCGGCAGCC<br>GCCCCCGGCACAGCCGCCCCCGCCGCGGCGCGGGGAAGTTTGGCCC<br>CGCGTGAGGATGCGGCTTTGTGTGTCCCCCCCTCAAATGCTCTTTGCT<br>TCGCTCCCAGCGCTCCGCGTACCCGAGAAAGCGGCAGCAGCGCACA<br>AAGCCCGGAGCTGCTCCCGATCCGTGCATGTGCTTTAGTTTGTGTCTG<br>TGTGTGTGTGTGTATGTGTGTGTGTGTGAGTGAGTGCGGTGTGTG<br>CGAGTGGCGTTTCTTGTTCTCTTGCAGGGTACAATGTTAAAAAGCCA<br>CCGCTAGTCGCCCCCAGTGCTCCTACTCTCTGGGTCTTTTTGTCTCTA<br>GTGCAGATTAAACGTCACGTCCGACGCGT

Note: The underlined letters are the mutation sites of IRF1, SMAD2, C/ EBP-β and GATA1, respectively.

Table S3. The primers information for overexpression, qRT-PCR

Genes/Plasmids	Primer Sequence (5′-3′)	Length/bp	NCBI Accession Number/gene ID
pcDNA3.1-IRF1	F:agcgtttaaacttaagcttggtaccATGCCCGTCTCAAGGATGC<br>R:aacgggccctctagactcgagTTACAAGCTGCAGGAGATGGCCT	942	NM_205415.2
pcDNA3.1-GATA1	F: **CGG**GGTACCATGGAGTTCGTGGCGCT<br>R:**CCG**CTCGAGTCAAATCTGCGGGCTCAG	915	NM_205464.1
ALV-J (*Env*)	F: GACTGGGCACCCTGGAAA<br>R: CCTGGGACAACGGAAATA	159	10206068
Bcl11b	F: GCACTGGTGGTGTCTGCTAT<br>R: TCTTTACCTGCAATGTTCTCCTG	187	XM_040702041.2
IRF1	F: GATCTCACGGCGAGGAAACA<br>R: AGCCATGCTTAGCTGCATGT	155	NM_205415.2
GATA1	F: CGATGCGCAAAGACGGAATC<br>R: CCTCCGGAGTTTCCAAAGGG	250	NM_205464.1
GAPDH	F: CGATCTGAACTACATGGTTTAC<br>R: TCTGCCCATTTGATGTTGC	151	NM_204305.2
SDHA	F: CAGGGATGTAGTGTCTCGT<br>R：GGGAATAGGCTCCTTAGTG	187	NM_001277398.1

Note: The lower-case letters are overlap sequences, underlined letters are enzyme cutting sites, bold letter is protective base.

Table S4. Target sequence of siRNAs

Genes	Name of siRNA	Target sequence
IRF1	siRNA1	GATCAACAAGGATAAGATGA
	siRNA2	AGCAAGAGAAAGTTGTATGA
	siRNA3	GCCATCTCCTGCAGCTTGTAA
GATA1	siRNA1	CGCAAAGACGGAATCCAAA
	siRNA2	CAGGCACAGTGTGCAGCAAC
	siRNA3	TGGGCAGACACGGGTACTTTGG
NC	Si-NC	CAAGCTGACCCTGAAGTTC

Table S5. Venn analysis of predicted transcription factors in different regulatory regions

Logic name set	Number	Transcription factor
–2999 ~ –2140 bp∩–2140 ~ –458 bp	12	AP-1, E2, Elf-1, GATA-1, GCN4, Hb, ICSBP, IRF-1, NF-kappaB, REV-ErbA, SRF, YY1
–2999 ~ –2140 bp	30	Ap-2alph, c-Ets-1, c-Fos, c-Jun, Cos, CPE bind, Dl, E4, E47, embryo D, Eve, GAL4, GLO, HNF-1, HNF-3, Ik-1, Ik-2, Ik-3, k-2a, Kr, MEB-1, MIG1, MyoD, NF-E2, Oct-2.1, PU.1, SRY, TBP, TEC1, Zen-1
–2999 ~ –2140 bp∩–2140 ~ –458 bp∩–458 ~ –281 bp	6	C/EBPα, NF-1, Oct-1, Sp1, Tra-1, USF
–2140 ~ –458 bp	27	Adf-1, ALF1B, AP-4, C/EBPγ, CACCC-binding, c-Myc, COUP, CPB bind, CREB, CRE-BP1, E1, EBF, ER, Erg-1, Ftz, GR, HNF-1C, Id3, JunD, LyF-1, MCM1, MRF4, NF-ATc3, NF-muE1, RAP1, RAR-β, RXR-β
–2140 ~ –458 bp∩–458 ~ –281 bp	6	AP-2, AP-2α, Egr-1, ETF, Krox-20, WT1
–458 ~ –281 bp	3	AP-2αA, NF-MuE1, N-Myc
–2999 ~ –2140 bp∩–458 ~ –281 bp	0	—
